# Bacterial translational regulations: high diversity between all mRNAs and major role in gene expression

**DOI:** 10.1186/1471-2164-13-528

**Published:** 2012-10-04

**Authors:** Flora Picard, Hélène Milhem, Pascal Loubière, Béatrice Laurent, Muriel Cocaign-Bousquet, Laurence Girbal

**Affiliations:** 1 Université de Toulouse; INSA, UPS, INP; LISBP, 135 Avenue de Rangueil, Toulouse, F-31077, France; 2 INRA, UMR792 Ingénierie des Systèmes Biologiques et des Procédés, Toulouse, F-31400, France; 3 CNRS, UMR5504, Toulouse, F-31400, France; 4 Institut de Mathématiques de Toulouse, UMR 5219, INSA de Toulouse, Université de Toulouse, 135 Avenue de Rangueil, Toulouse, F-31077, France

**Keywords:** Translational regulation, mRNA Ribosome, Translatome, Statistical modeling, *Lactococcus lactis*

## Abstract

**Background:**

In bacteria, the weak correlations at the genome scale between mRNA and protein levels suggest that not all mRNAs are translated with the same efficiency. To experimentally explore mRNA translational level regulation at the systemic level, the detailed translational status (translatome) of all mRNAs was measured in the model bacterium *Lactococcus lactis* in exponential phase growth.

**Results:**

Results demonstrated that only part of the entire population of each mRNA species was engaged in translation. For transcripts involved in translation, the polysome size reached a maximum of 18 ribosomes. The fraction of mRNA engaged in translation (ribosome occupancy) and ribosome density were not constant for all genes. This high degree of variability was analyzed by bioinformatics and statistical modeling in order to identify general rules of translational regulation. For most of the genes, the ribosome density was lower than the maximum value revealing major control of translation by initiation. Gene function was a major translational regulatory determinant. Both ribosome occupancy and ribosome density were particularly high for transcriptional regulators, demonstrating the positive role of translational regulation in the coordination of transcriptional networks. mRNA stability was a negative regulatory factor of ribosome occupancy and ribosome density, suggesting antagonistic regulation of translation and mRNA stability. Furthermore, ribosome occupancy was identified as a key component of intracellular protein levels underlining the importance of translational regulation.

**Conclusions:**

We have determined, for the first time in a bacterium, the detailed translational status for all mRNAs present in the cell. We have demonstrated experimentally the high diversity of translational states allowing individual gene differentiation and the importance of translation-level regulation in the complex process linking gene expression to protein synthesis.

## Background

Variations in protein concentrations are not solely related to transcriptional activity. This is also true in bacteria. Recent studies with *Escherichia coli* and *Desulfovibrio vulgaris* described only moderate correlations between mRNA levels and protein concentrations with a Pearson coefficient varying from 0.45 to 0.53 according to culture conditions [1,2]. In *Lactococcus lactis,* a gram positive lactic acid bacterium, the Pearson coefficient was even lower ranging from 0.19 to 0.24 [3]. Post-transcriptional events (*e.g.* protein stability and translation regulation) are proposed to be the principle causes of these weak correlations [4]. In *L. lactis*, we identified protein abundance determinants by statistical modeling of proteome data: gene sequence features such as the Codon Adaptation Index (CAI), gene length and functional categories were demonstrated to strongly influence mRNA translation levels [3]. In addition, individual mRNA translation efficiency was estimated by a mechanistic modeling approach, highlighting significant variability in translation efficiencies among the different genes of the genome [5]. Consequently in *L. lactis*, not all mRNAs are believed to be translated with the same efficacy and important regulations are expected to occur at the translational level.

An experimental approach for studying genome scale translational regulation is translatome analysis which provides the detailed translational status of all mRNAs. Translation consists of three steps: initiation, elongation and termination. Translation initiation occurs with a ribosome binding to the mRNA template and begins at the start codon with incorporation of the first amino acid of the polypeptide. The ribosome continues to translate the coding sequence catalyzing polypeptide chain elongation until it reaches the stop codon. Then, translation termination occurs and the complete polypeptide chain is released. Translation is carried out by more than one ribosome simultaneously forming a polysomal structure. Translatome experiments provide the ribosome number on each mRNA molecule in the cell. Translatome determination combines size separation of ribosome-mRNA complexes according to the number of ribosomes loaded (polysome profile) and measurement of mRNA levels in fractions by microarray techniques [6]. Most translatomes reported so far aimed at identifying translationally regulated genes in response to stress [7,8] or changing growth phase [9]. However, these studies were based on low-resolution polysomal profile analysis in which messengers were classified into only two fractions: polysomal *versus* non-polysomal (the polysomal fraction corresponding to the strongly-translated mRNAs loaded with several ribosomes, and the non-polysomal one including weakly or untranslated mRNAs). In these conditions, the cellular translational status of each mRNA molecule was not fully described since the number of loaded ribosomes was not quantified. However, this information is required to study the diversity of translational regulations between all mRNA species present in a cell and then to understand translation efficiency of individual mRNAs. To date, only a few studies described high resolution translatome analysis in microorganisms with mRNAs classified with respect to the precise number of loaded ribosomes [10,11]. Unfortunately all high resolution translatome studies were carried out in yeast, leaving the understanding of detailed translational regulation in bacteria incomplete.

Therefore we present here the first high resolution translatome analysis in the bacterium *L. lactis*. This model lactic acid bacterium is a gram-positive non spore forming bacterium with a low GC content (35%). *L. lactis* is therefore phylogenetically closer to *Bacillus subtilis* than *E. coli. L. lactis* was grown under maximum growth rate conditions (exponential phase). The profile of mRNA-ribosome association led to the definition of two translational variables for each mRNA species: the fraction engaged in translation (ribosome occupancy) and the ribosome density. By comparing the translational states of all mRNAs, the regulation of both ribosome occupancy and ribosome density levels were explored. In addition, the influence of ribosome occupancy and ribosome density on the final protein expression level was quantified. This demonstrated the key role of the mRNA translational status in the complex processes linking gene expression to protein synthesis.

## Results

### Polysomal profile description

**Figure 1:**
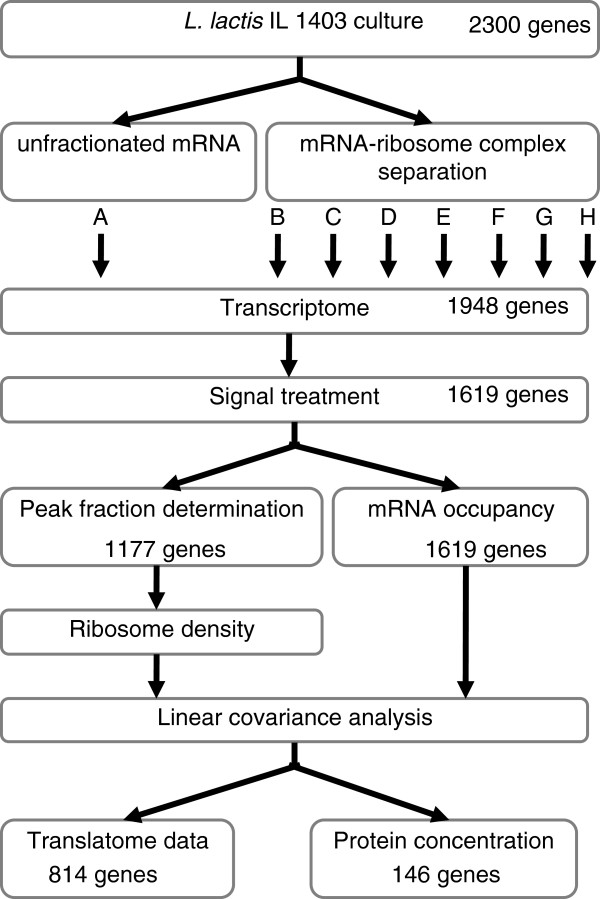
**Schematic overview of the translatome analysis in exponential phase *L. lactis *cells.** For each step, the size of the gene set is provided.

**Figure 2:**
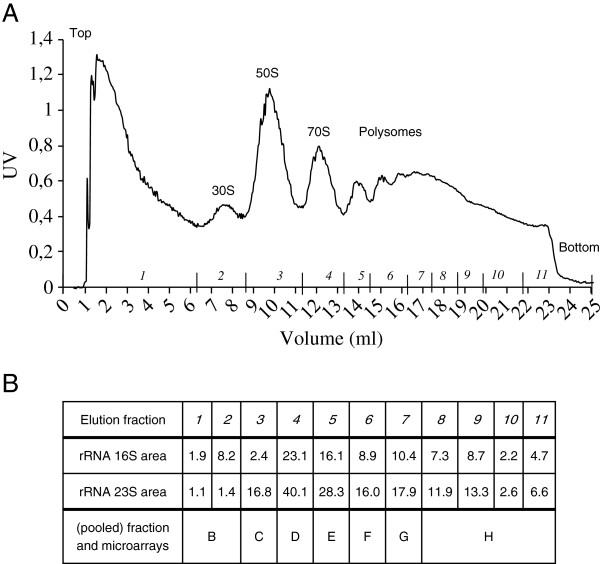
**Polysomal profile of *L. lactis *cells in exponential phase**. (**A**) 254 nm absorbance profile. The top and bottom of the gradient were indicated on the left and right of the profile, respectively. (**B**) 16S and 23S rRNA quantifications for peak assignment.

**Figure 3:**
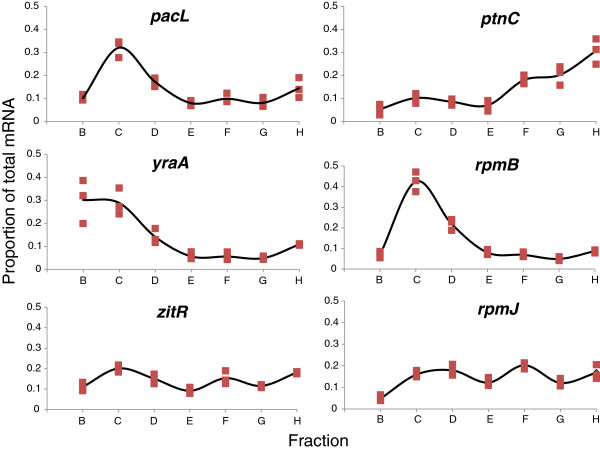
**mRNA proportions between fractions B to H for six chosen genes.** For a given gene, its proportions of mRNA molecules in each fraction from **B** to **H** were calculated as described in Equation 6 of Methods. Square symbols represent data from the three polysomal profile experiments. The black line is the plot of the mean mRNA proportion value.

The translatome of *L. lactis* was studied by coupling polysome profile determination, transcriptomics and statistical analyses (Figure 1). A typical polysome profile is shown in Figure 2. After peak assignment, pooling of the two first fractions corresponding to mRNAs not engaged in translation and pooling of the last four fractions representing the most highly ribosome-loaded transcripts, the seven resulting fractions were hybridized to the microarrays B to H, respectively (Figure 2). 1619 genes were selected according to the cutoff criterion (see Methods). Figure 3 shows sample distributions of mRNA proportion between fractions B to H for six selected genes. When considering the entire gene set, 61% and 37% of genes exhibited the highest mRNA abundance in fraction C (18 to 42% abundance range with a mean value of 25 ± 4%) or in fraction H (18 to 34% abundance range with a mean value of 23 ± 3%), respectively.

The number of ribosomes per transcript was one for fraction D, and in the range 1.4-2.9 (mean value 2.1), 2.9-5.4 (mean value 4.1), 5.4-9.6 (mean value 7.4) and 9.6-17.9 (mean value 14) for fractions E, F, G and H, respectively (see Methods). A maximum of 18 ribosomes per transcript was thus obtained in *L. lactis* cells grown in the exponential phase. The percentage of ribosomes engaged in translation was estimated by area integration of the polysomal profile (see Methods) and an average ratio of 61 ± 2% of total ribosomes engaged in translation was obtained. Since about one third of the total ribosomal content was not associated with mRNA, ribosomal content would appear in excess for protein synthesis under the growth conditions used in this study.

### Translatome variable determination and physiological analysis

**Figure 4:**
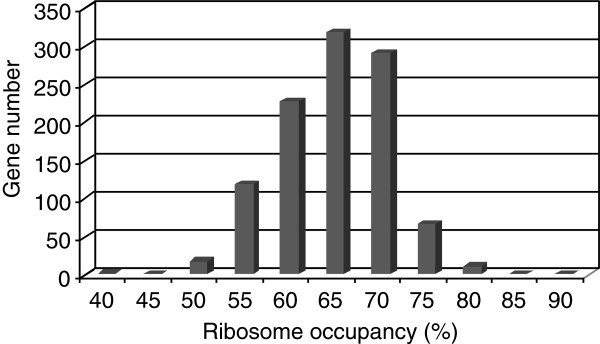
**Ribosome occupancy distribution**. The percentage of each mRNA found in ribosome-containing fractions (fractions D to H) was determined for 1619 genes.

Two translatome variables were determined, the ribosome occupancy corresponding to the fraction of mRNA engaged in translation and the ribosome density, the number of loaded ribosomes per 100 nucleotides. Ribosome occupancy reflects the efficiency of translation initiation while the interpretation of ribosome density can vary according to the limiting step of translation. For genes exhibiting initiation-limited translation, high density is correlated with high translation levels, but for genes with elongation limitation, high density can result from ribosome congestion leading to low translation efficiency. Ribosome occupancy values were obtained for 1619 genes, and a Gaussian distribution was observed with a median value of 66 ± 6% (Figure 4). High variability in ribosome occupancy was observed among genes with values ranging from 41% to 84%. Therefore, for all genes at least part of the corresponding mRNA molecules was involved in translation but never the entire mRNA population. This suggests that for individual genes, the mRNA concentration in exponentially-grown cells of *L. lactis* was in excess. Thus under the conditions used, both ribosome availability (see above) and mRNA abundancies could conceivably support higher protein synthesis.

A peak fraction, corresponding to the most frequently found number of ribosomes bound on each mRNA, was assigned to 1177 genes with a 95% bootstrap confidence interval. Except one gene (encoding the ribosomal protein RpmJ) with a peak fraction in fraction F (2.9-5.4 loaded ribosomes), all the other genes grouped into two classes of the peak fraction. The first class was composed of 200 genes translated with a low number of ribosomes with a peak fraction in the monosome fraction D. The second class included 976 more highly loaded genes with a peak fraction in fraction H, corresponding to 9.6-17.9 loaded ribosomes. We verified that the bacterial specificity of polycistronic structures did not introduce any bias in ribosome number determination. In *L. lactis,* 38 genes have been experimentally confirmed in the literature as being organized as polycistronic operons [12-15]. They were specifically examined to validate their eventual enrichment in the two subgroups of genes with peak fractions in D and H, respectively. No significant over-representation of these genes was observed.

**Figure 5:**
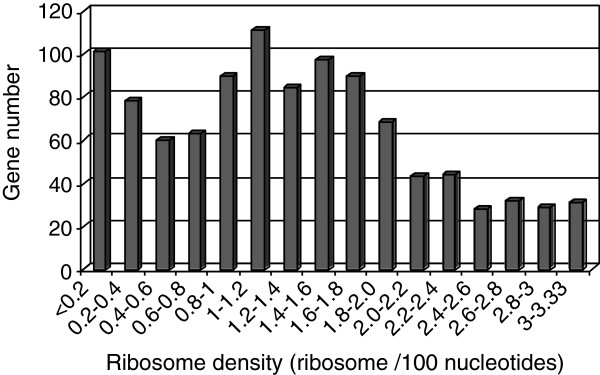
**Ribosome density distribution**. The ribosome density (expressed as ribosomes per 100 nucleotides) was determined for the set of 1049 genes.

Based on the peak fraction value, the second translatome variable, ribosome density, was calculated. For each of the 1177 genes, ribosome density was assessed as the ratio of the ribosome number in its peak fraction to the coding sequence length. If we consider that one bound bacterial ribosome protects around 30 nucleotides [16], the theoretical maximal ribosome density is 3.33 ribosomes /100 nucleotides. However, 128 genes within the 1177 gene set exhibited experimental ribosome densities higher than 3.33. This 128 gene subgroup was significantly enriched in short monocistronic genes (with 118 gene lengths < 400 bp, *p*-value <10^-20^). The presence of rather long 5’UTRs (up to several hundred bases in prokaryotes [17]) could contribute to ribosome density over-estimation, even more pronounced in the case of short genes. This set of 128 genes with aberrant ribosome densities was omitted in the subsequent analyses leading to a new set of 1049 genes. Figure 5 shows the distribution of ribosome density ranging from 0.02 to 3.31 ribosomes per 100 nucleotides. The median ribosome density was 1.23 ribosomes per 100 nucleotides (mean value: 1.31 ribosomes per 100 nucleotides) representing 2/5 of the maximal theoretical ribosome density. In *L. lactis,* for most of the genes, ribosome density was thus far from the maximal density as also reported in yeast [10,11]. Therefore, as proposed in yeast [11], translation limitation appears to occur during ribosome loading on mRNA in the initiation step even though ribosomes and mRNA molecules were found in excess in *L. lactis* cells. With a strictly mathematical modeling approach of translation control such low ribosome densities were previously demonstrated in bacteria to be related to a limitation of translation at the initiation step [18,19]. Thus in this context of initiation-limited translation, high ribosome density is expected to provoke high translation levels for most of the *L. lactis* genes.

Homolactic lactic acid bacteria such as *L. lactis* are fermentative bacteria producing high concentrations of lactate during glucose catabolism (a conversion yield of ~90%). The lactate production ensures cofactor (NADH) regeneration which is essential for bacterial activity but also induces the acidification of the ambient environment and thereby inhibition of bacterial growth [20]. Genes involved in important metabolic functional categories exhibited similar regulation of ribosome density and ribosome occupancy (Additional file 1: Figure S3). In agreement with the high growth rate, *L. lactis* favoured the translation of specific functions required for optimal growth. A high glycolytic rate was favoured by the efficient translation of the first step of the glycolysis, the glucose transport system, which is believed to control the glycolysis rate in the studied IL1403 *L. lactis* strain [21]. High ribosome occupancy and ribosome density were indeed observed for the main glucose transport system, mannose PTS (*ptnABCD*) and glucokinase (*glk*), which are required when glucose enters through sugar permease. The sugar permease system is still uncharacterised in *L. lactis*, however our finding of a high level of translation (both translatome variables above the average) of the gene *ypcH* coding for a sugar permease suggests the involvement of this permease in glucose transport. Similarly, the last step of glycolysis, corresponding to conversion of pyruvate into lactate by lactate dehydrogenase (*ldh*), also corresponded to a very efficiently translated gene (with high ribosome density and ribosome occupancy of 1.43 and 78%, respectively). In order to counteract growth inhibition by cytoplasmic acidification under conditions of extracellular auto-acidification, translation was also increased for almost all genes coding for the ATPase system (*atpBEFGH*) that catalyses the excretion of protons while consuming ATP. This proton expulsion system was efficient enough to prevent a strong acid stress since secondary pathways of the acid stress response were not favoured at the translational level. Genes of the arginine deiminase and glutamate decarboxylation pathways (*arcC2D1D2* and *gadB,* respectively) exhibited low values of ribosome occupancy and ribosome density in comparison to the average. In addition, positive regulation of translation of enzymes related to phosphate limitation (regulator *phoL* and the high affinity transporters *pstABC* and *phnC*), and to zinc and manganese transports (*zitR*, a zinc transport regulator and the manganese transporters *mtsABC*) were observed, suggesting additional cation and anion requirements to fight against lactic acid inhibition.

### Determinants of translatome variables

In order to identify how ribosome occupancy and ribosome density levels can be regulated, we searched for their major biological determinants. First, sequence analysis was performed to identify DNA patterns which could be specifically associated with genes with high or low levels of ribosome occupancy or ribosome density, respectively. We selected short nucleotide sequences (55 bp) in the vicinity of the ribosome binding site and larger sections (125 bp) containing the entire 5’UTR (defined in previous work; [22]). According to their ribosome occupancy level, genes were first grouped into two sets containing the 205 genes with the highest (> 0.72) and the lowest (< 0.60) values, respectively. In the nucleotide region from −30 to +24 relative to the start codon, we did not detect any discriminating sequence motif. With enlarged nucleotide sequences (from −100 to +24), we detected, for high ribosome occupancies exclusively (first quartile), a conserved DNA pattern of 8 nucleotides whose sequence was A[CA]TGACAG. The *E*-value of this pattern calculated with MEME software (4.1 x 10^-46^) was significantly lower than that of the sequence GGAGG, also identified as a conserved sequence in this subset of genes (*E*-value of ~10^-15^). In *L. lactis*, when considering all gene sequences (−30 to +1 bp relative to the start codon) the GGAGG motif showed a *E*-value of 6.1 x 10^-59^, confirming its role as a Shine-Dalgarno sequence by base-pairing with its complementary sequence in the 3’end of *L. lactis* 16S rRNA molecules. 31 genes of the high ribosome occupancy set had the A[CA]TGACAG sequence generally located upstream of AUG, and 18 presented repeated patterns, including some inverted repeats suggesting formation of hairpin structures. Concerning the ribosome density level, no conserved DNA pattern was found in the enlarged nucleotide region with genes of the first or fourth quartiles.

In order to identify other key systemic factors of translation, we used a more global approach and we statistically quantified the influence of various parameters related to gene, mRNA and protein features on the two translatome variables. All genome-scale data (transcriptome [5], mRNA half-lives [22], and translatome (this work)) were obtained for *L. lactis* cells grown in identical standardized conditions in bioreactors to avoid introducing experimental artifacts linked to minor changes in growth conditions. These omic data are indeed very sensitive to bacterial adaptation to changing conditions. We used the statistical method of parameter selection developed previously [3]. With this approach, we identified and classified the most significant parameters which could explain the different levels of translatome variables observed between all mRNAs without any *a priori* subjective selection. Parameters included in the models were features related to gene (CDS length, CAI, chromosomal position and functional category), to mRNA (concentration, half-life and folding) and to protein sequence (aromatic and hydrophobic properties). For this statistical modeling approach, the gene data set was reduced from 1049 to 814 due to undetermined parameter values, mainly in the mRNA half-life data set.

Models of ribosome occupancy and ribosome density were obtained with adjusted squared-r equal to 0.34 and 0.22, respectively (Table 1). Significant parameters were selected for both variables, and results were confirmed by simple linear regression analysis (Additional file 2: Table S2). First, mRNA half-lives had a moderate negative estimated coefficient with both translatome variables, with a *p*-value < 2.0 x 10^-16^ (Table 1). The ribosome occupancy and ribosome density were also positively related to the folding energy in the vicinity of the start and stop codons of mRNA (positive correlation coefficients with ΔGup and ΔGdown, predicted minimum free energy of mRNA structures in upstream sequences and in downstream sequences, respectively) (Table 1). These results indicated that less stable mRNAs and mRNAs with less stable secondary structures in 5’ and 3’UTR regions could be better translated with higher level of ribosome occupancy. Furthermore, for the majority of the *L. lactis* genes translationally limited at the initiation step, increased ribosome density could also reflect higher level of translation. Two interpretations can be proposed for the striking inverse relationship between mRNA stability and both translatome variables. Since the most stable mRNAs were shown in *L. lactis* to be those present at low concentrations [22], we can hypothesize that the low ribosome occupancy of stable transcripts could result from a low meeting probability between low concentrated mRNA molecules and ribosomes. However, in our 814 gene set, the top 10% less concentrated mRNA displayed, as expected, increased stability compared to the average (+77%), but their ribosome occupancy was only slightly decreased (−5%). A more probable interpretation would be related to the presence of a specific pattern in the group of genes with high mRNA stability and low translational variables. We have previously reported in *L. lactis* that an over-representation of the purine-rich sequence AGGAG was present, as in *Bacillus*, in the 5’UTR of stable transcripts [22]. A hypothesis could be that this sequence blocks translation by inducing ribosome stalling. For 13 genes of our 814 gene set having more than one AGGAG sequence in their 5’UTR (11 genes with two AGGAG and two genes with three repeats), we observed, indeed, a stabilization of the transcript (+34%) compared to the average, associated with a significant reduction (−33%) of the ribosome density.

**Table 1:** Estimated coefficients of ribosome occupancy and ribosome density models

**Parameter**	**Variable to explain: Ribosome occupancy**	**Variable to explain: Ribosome density**
mRNA concentration	0.12 (*p* = 1.2 x 10^-4^)	−0.15 (*p* = 4.0 x 10^-6^)
mRNA half-life	−0.38 (*p* < 2.0 x 10^-16^)	−0.36 (*p* < 2.0 x 10^-16^)
CDS length	0.24 (*p* = 2.3 x 10^-14^)	−0.22 (*p* = 1.4 x 10^-10^)
Aromaticity	/	0.09 (*p* = 8.7 x 10^-3^)
CAI	0.16 (*p* = 6.3 x 10^-6^)	−0.11 (*p* = 7.6 x 10^-3^)
ΔGdown	0.12 (*p* = 4.0 x 10^-5^)	0.12 (*p* = 3.0 x 10^-4^)
ΔGup	0.15 (*p* = 4.0 x 10^-7^)	0.15 (*p* = 5.7 x 10^-6^)
Functional category AMI	/	−0.20 (*p* = 5.0 x 10^-3^)
Functional category COF	/	−0.35 (*p* = 4.4 x 10^-2^)
Functional category INT	0.68 (*p* = 1.6 x 10^-3^)	/
Functional category REG	0.62 (*p* = 1.7 x 10^-5^)	0.45 (*p* = 3.9 x 10^-3^)
Functional category TRS	−0.23 (*p* = 4.0 x 10^-2^)	/
Adjusted R^2^	0.34	0.22

*ΔGdown* and *ΔGup*: free energy of the predicted minimum free energy structure for upstream mRNA sequences (from −100 to +24 bp relative to the start codon) and downstream sequences (from −24 to +100 bp relative to the stop codon), respectively. *AMI*: amino acid biosynthesis, COF: biosynthesis of cofactors, *INT*: central intermediary metabolism, *REG*: regulatory functions, *TRS*: transcription.

While the previous parameters exhibited additive influence on both translatome variables, some parameters lead to antagonistic effects. The length of the coding sequence had a negative coefficient for ribosome density (Table 1) and this conclusion was valid for the two main classes of mRNAs (with low and high loaded-ribosome numbers) (Additional file 3: Figure S4). In contrast, CDS length had a positive coefficient for ribosome occupancy. Since translation of most *L. lactis* genes is expected to be initiation-limited, higher ribosome density of small mRNAs could counteract their lower proportion involved in translation. Similarly, mRNA concentration and also CAI showed a positive coefficient for ribosome occupancy (0.12 and 0.16, respectively) but were negatively correlated with ribosome density (−0.15 and −0.11, respectively). CAI is assessing translation elongation efficiency as a function of genes’ codon usage. This statistical result confirms what is generally assumed. For genes with elongation-limited translation, high CAI values correspond to fast translation and therefore low ribosome density. Protein amino acid composition also interfered with the translation process but only at the ribosome density level.

Our modeling approach also allowed to analyze the relationship between gene function [23] and translatome variables. Genes involved in regulatory functions possessed higher ribosome occupancy and ribosome density than the average (positive coefficients of 0.62 and 0.45 in ribosome occupancy and ribosome density models, respectively). These regulatory genes coded mainly for transcriptional regulators involved in specific metabolisms (*e.g.* arginine (*ahrC*), pyrimidine (*pyrR*), biotin (*birA1*) or sugar (*lacR, citR*)) but also for general transcriptional regulators (*codY* and *ccpA*) and central regulatory protein (*relA*). On the other hand, genes related to synthesis of both amino acids and cofactors exhibited significantly lower ribosome density than the other functional categories.

### Translatome variables as determinants of protein concentration

**Figure 6:**
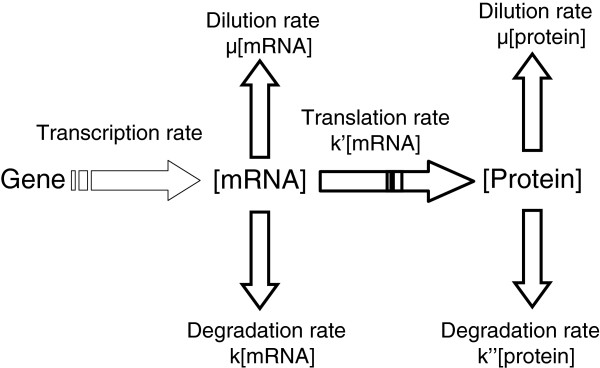
**Modeling of the cellular process.** Genes are first transcribed into mRNA before being translated into proteins. Proteins are then submitted to dilution by growth and degradation. μ: growth rate; k: mRNA degradation constant; k': translation efficiency; k'': protein degradation constant.

Next we aimed to determine the influence of translational regulations downstream in the gene expression process. More particularly, we analyzed protein concentration variations in relation with their mRNA ribosome occupancy and ribosome density values. Protein level is generally considered as the final result of translation. The amount of protein present results from complex multilevel regulations, some being independent of translation (Figure 6). Thus, protein concentrations are difficult to predict [2,24]. In *L. lactis*, some determinants of protein levels were identified by statistical modelling but without taking into account any translational parameters [3]. Here, we added the two translation variables to the previously analyzed parameters to quantify the involvement of translation in the control of protein levels. We took again special care to use proteomic data [5] acquired in *L. lactis* under our standardized conditions (exponential phase). Due to the low number of proteins identified in this growth condition, the data set was reduced to 146 genes. Our understanding of protein concentration determinants was improved upon inclusion of the translatome data (Table 2). The adjusted squared-r of the model slightly increased with the translatome data from 0.52 to 0.56. However, we observed a key influence of the ribosome occupancy on protein levels, with a positive estimated coefficient of 0.23. Like in the original model without translational parameters, CAI (estimated coefficient of 0.54) and mRNA concentration (estimated coefficient of 0.25) also made a significant contribution to the protein level (Table 2).

**Table 2:** Selected determinants of protein level covariance models

**Parameter:**	**Variable to explain: protein level**	
	**Model (I)**	**Model (II)**
mRNA concentration	0.33 (*p* = 3.0 x 10^-7^)	0.25 (*p* = 9.7 x 10^-5^)
CAI	0.54 (*p* = 3.5 x 10^-15^)	0.54 (*p* = 1.9 x 10^-14^)
Ribosome occupancy	/	0.23 (*p* = 5.4 x 10^-4^)
Adjusted R^2^	0.52	0.56

Model (I) is the original model from [3]. Model (II) contains the parameters as Model (I) plus two new explanatory parameters, ribosome occupancy and ribosome density.

## Discussion

In this study we have provided, for the first time, a detailed and complete picture of translation in a bacterium. Translatome experiments allowed the determination of the translational status of each mRNA including its ribosome occupancy and ribosome density. In the bacterium under study, *L. lactis*, the fraction of mRNAs engaged in translation was on average 67% and the mean ribosome density reached 2/5 of the maximal theoretical density. Similar results have been obtained in yeast [10,11], although notable differences in the translation process itself (localization, coupling with transcription) and in its regulation (RBS, translation factors) are present between yeast and bacteria. Our data and data obtained in yeast [10,11], show that low ribosome density and incomplete mRNAs engaged in translation are two general translational rules in both kingdoms. The low ribosome density indicated for most *L. lactis* genes that translation is limited at the initiation step. In this particular case, higher ribosome density would result in higher translation level. We have demonstrated that the two translational variables, ribosome occupancy and ribosome density were not constant for all the genes examined here. A high level of variability was indeed demonstrated within the mRNA population, indicating that even if global translation seems to be conserved between microorganisms, this process is also highly transcript-specific. We showed in addition that genes involved in key metabolic pathways exhibited coordinated regulation of ribosome density and ribosome occupancy levels. This result suggests the possibility for *L. lactis* to use fine translation tuning of selected transcripts during its adaptation to the external environment (*e.g.* conditions of optimal growth rate). We have also demonstrated that ribosome occupancy was a major determinan of protein level, revealing the high influence of translational regulations in the process coupling gene expression to protein synthesis.

By exploring the diversity of ribosome occupancy and ribosome density values within the mRNA population, systemic key factors involved in translation level regulation were sought. The systemic influence of some factors (codon usage, secondary structure…), previously identified as protein expression regulators of specific mRNAs [25,26] or predicted as determinants of translational efficiency by transcriptome-proteome correlation analyses [2,27,28] was seen to act directly on translatome variability. In addition, less expected general factors influencing translation, such as gene function, mRNA half-life and gene length were identified and organized hierarchically. Contribution of these parameters to differentiated translational regulation in natural and stress conditions was also reported in yeast [10,11,29,30]. Linear covariance models established in *L. lactis* a link between translational efficiency and gene function. First, genes involved in biosynthesis of amino acids and cofactors exhibited lower ribosome density than others. *L. lactis* thus adapted its metabolism by attenuating translation of genes involved in these two anabolic pathways. The chemically defined medium used in this study for growing the bacterium contains all the amino acids and vitaminic precursors of the cofactors. In these growth conditions, *L. lactis* thus adapted its metabolism to ensure cofactor and amino acid supply via their import from external medium rather than via *de novo* synthesis. This translational attenuation constitutes an adaptive response appropriate to limit energy wastage in the bacterium. We have also observed high ribosome occupancy and high ribosome density for genes of the functional category “regulatory functions”. Important transcriptional regulators were concerned suggesting their efficient translation *in vivo*. This finding illustrates the capacity of *L. lactis* cells to coordinate the control of the entire gene expression network through positive translational regulations of key regulators. Mechanisms of transcriptional control by translation have already been reported in *E. coli*. For mRNAs of some amino acid metabolic enzymes, ribosome deceleration leads to attenuation or antitermination of transcription [31,32]. In a more general way, ribosome acceleration and deceleration were shown to directly result in corresponding changes in speed of the RNA polymerase [33].

In addition, an inverse relationship between mRNA stability and both translatome variables was found. As a consequence, less stable mRNAs tended to exhibit rather efficient translation initiation. For initiation-limited unstable genes, higher ribosome density would lead to higher level of translation. mRNA decay and translation would thus act in an antagonistic manner on gene expression regulation in *L. lactis.* These results are in contrast with what was observed in yeast [11]. A positive relationship between ribosome density and mRNA half-life was expected in *L. lactis*, assuming the protective effect of ribosomes against RNases. The absence of such a positive correlation indicates that protection of mRNA against degradation should be more related to ribosome position than to ribosome density. In *B. subtilis*, mRNA protection by ribosomes located far upstream of cleavage sites (without direct shielding of these sites as required in *E. coli*) was previously demonstrated [34].

Translation in *L. lactis* was strongly dependent on coding sequence length, ribosome density being negatively correlated with gene length. Such an inverse correlation between ribosome density and gene length was also reported in yeast [10,11]. It would be interesting to verify if an excess of ribosomes at the beginning of the coding sequence as reported in yeast [35,36] is also present in *L. lactis* to explain why short mRNAs tended to present higher ribosome density. However, contrary to yeast [11], a positive correlation was obtained between ribosome occupancy and gene length. This correlation is probably necessary in bacteria to counteract the negative ribosome density effect in order to not penalize translation of long important mRNAs. In *L. lactis* the longest genes represent major functions involved for example in replication, energy metabolism or transport (enrichment results not shown). But more generally, in bacteria, rather long mRNAs are expected in comparison to yeast as a consequence of numerous polycistronic structures.

Using our translatome data, the genome-scale regulation of the initiation and elongation steps was analyzed. mRNA folding was identified as a key determinant of ribosome occupancy. Site-directed experiments at the initiation region have previously demonstrated in *E. coli* and in *L. lactis* the association between mRNA folding and translation initiation efficiency [37,38]. mRNA folding is expected to interact with ribosome binding site accessibility and modify individual gene expression as demonstrated in *E. coli*[25,39]. In addition, for genes with high ribosome occupancy, we have identified a conserved nucleotide pattern (sometimes forming inverted repeat sequences) upstream of the start codon. It is thus tempting to speculate that these sequence features could modulate ribosome binding. Specific translation-enhancing sequences upstream of the start codon have been reported in *E. coli* and proposed to act as additional interaction sites with either 16S rRNA or S1 ribosomal protein [40,41]. However, *L. lactis* 16S rRNA does not contain a complementary sequence to our motif. In addition, as in other gram-positive bacteria [42], no homologous protein of the ribosomal S1 protein was found in *L. lactis*. Thus, mechanisms of enhancement of translation initiation based on improved mRNA-rRNA base pairing or on increased ribosomal protein-mRNA interaction seem unlikely in *L. lactis*. Alternatively, the conserved nucleotide pattern found in *L. lactis* could be involved in secondary structure stability playing a role in translation initiation efficiency [38]. In yeast, specific base usages around the start codon for genes with high ribosome occupancy were also reported [28]. Here two determinants of the initiation step were identified; they could be thus involved in the initiation limitation of translation of most of *L. lactis* genes. Nevertheless, two factors of the elongation step, codon usage and amino acid composition, were shown to influence translation in *L. lactis* (both at the level of ribosome density). CAI is widely accepted as the key factor in the determination of the elongation rate [4,27]. Therefore, our results indicate that for some genes the elongation step could also contribute to translation control corroborating the current point of view of mixed control of translation by initiation and elongation [4,19,27,28,36].

To further explore translation regulation, local mRNA ribosome density could be explored via ribosome density mapping or ribosome footprint experiments [43-45]. Comparison of ribosome density in the 5’UTR of mRNAs exhibiting high or low ribosome occupancies could help to confirm the link between the sequence signatures identified in this work and efficient ribosome binding. The determination of local ribosome densities within a coding sequence could contribute to a better understanding of the relationship between ribosome density and translation efficiency.

## Conclusions

This study described a global analysis of translation-level regulation in a model bacterium (*L. lactis*)*.* Studies describing the detailed translational status of an entire mRNA population (translatome data) are rare and were until this work only available in the yeast model. For each mRNA of *L. lactis* in the exponential growth phase, we have determined the number of ribosomes loaded as well as the proportion of mRNA involved in translation. A high variability in the translational status was demonstrated within the mRNA population reflecting transcript-specific translational level regulation. Integration of translatome data with other relevant omic data (proteome, transcriptome, mRNA half-lives…) using statistical modeling let to the identification of key regulatory parameters required for efficient translation. This approach significantly increased the knowledge of translational control in microorganisms and concurred in the understanding of how post-transcriptional regulation leads to weak correlations between transcriptomic and proteomic data. The main conclusion of our work was that translational regulation plays, as transcriptional regulation, an important role in the control of protein expression levels.

## Methods

### Organism and growth conditions

*Lactococcus lactis* subsp. *lactis* IL1403 was grown under anaerobic conditions in batch cultures at 30°C, pH 6.6 and 250 rpm, in a chemically defined medium as previously described [22].

### Polysomal RNA preparation

Polysome profile determination was adapted from the protocol developed in yeast [6]. Cells were cultivated in exponential phase at a maximum growth rate of 0.88 h^-1^ to an optical density of 1 at 580 nm. Translation elongation was then arrested by adding 100 mg/ml chloramphenicol and cells were collected on ice. A total amount of 96 mg of dried cells was harvested at 4°C (3645 g, 8 minutes), resuspended at 4°C in lysis buffer (20 mM Tris HCl pH 8, 140 mM KCl, 40 mM MgCl_2_, 0.5 mM DTT, 100 μg/ml chloramphenicol, 1 mg/ml heparin, 20 mM EGTA, 1% Triton X-100) and washed twice. Then, the cells were disrupted at 4°C in tubes containing 0.1 g of glass beads using Fast Prep ® (4 cycles of 30 seconds at 6.5 m/sec with 1 minute intervals). After centrifugation, the supernatant was loaded onto a 24-ml linear sucrose gradient (10 to 50% (w/v)) in polysomal gradient buffer (same composition as lysis buffer except for heparin at a final concentration of 0.5 mg/ml). Polysomal complexes were resolved by centrifugation at 13,500 rpm for 16 h 30, at 4°C in an SW 28 rotor. mRNA-ribosome complexes were separated in 11 elution fractions eluted in cold buffer (55% sucrose (w/v), 500 mM Tris HCl pH 8, 10 mg/ml Bromophenol blue) at a speed of 2.5 ml/minute. Absorbance at 254 nm was measured continuously with a UV detector (UPC900 Amersham Pharmacia Biotech). In each fraction, a protein elimination step was performed by adding one volume of 8 M guanidium-HCl and two volumes of absolute ethanol [6]. RNAs were extracted with the Qiagen Rneasy Midi kit®. Peak assignment for ribosomal subunits, monosomal and polysomal complexes, was achieved through 16S and 23S rRNA measurements in each elution fraction by RNA analysis using Bioanalyzer (Agilent technologies®). Peak assignment identified 30S and 50S ribosomal subunits in elution fractions 2 and 3, respectively (Figure 2). Consequently, elution fraction 1 corresponded to free RNAs, while eluted fractions above number 3 contained mRNAs associated with at least one complete ribosome. To reach the 5 μg of total RNA required for transcriptome analysis, the two fractions eluted first and the last four fractions were pooled, respectively (Figure 2).

Three independent experiments from culture to polysome profile determination were carried out. An aliquot of cell-free extract was used in parallel to provide unfractionated total RNA (sample named A). Protein elimination and RNA extraction were performed as described above. This unfractionated RNA was used as an internal reference for normalization (see below) and to check the reproducibility of polysome separation. To avoid mRNA degradation, the fractionation experiment was performed at 4°C and inhibitors of ribonucleasic activity were added in buffers. A loss of mRNA was however observed but this loss was constant between genes and repetitions. The percentage of mRNA molecules recovered after the polysome separation compared to unfractionated RNA was per gene in average at 60 ± 16%.

### Ribosome number extrapolation

For each polysomal profile, the number of ribosomes per transcript in the fractions lacking one single ribosome resolution (fractions F to H) was estimated by a logarithmic extrapolation from the monosome peak (fraction D) with the following Equation [46]:

(1) $$\text{ln}\left(Elution_{\mathrm{time}}\right)=a\times \text{ln}\left(P_{\mathrm{size}}\right)+b$$

with the *a* slope coefficient corresponding to the averaged value of the three repetitions and the *b* constant related to the elution time of the monosome fraction specific of each repetition.

### Ribosome engaged in translation

The percentage of ribosomes engaged in translation was estimated by area integration of the 254 nm absorbance of the polysomal profile. The ratio of the area under the absorbance curve corresponding to translating ribosomes (elution fractions 4 to 11) over the area corresponding to total (translating and non-translating) ribosomes (elution fractions 2 to 11) was calculated for the three polysomal profiles.

### Transcriptomic analysis and normalization

For signal normalization and modeling, the free statistical software R ( http://www.r-project.org/) was used.

Gene expression was measured using nylon arrays containing PCR fragments (Eurogentec®) for 1948 genes of *L. lactis* IL1403. Nylon array spotting and analytical support were provided by the Biochips Platform (Genopole Toulouse, France). From each fraction from B to H and from the unfractionated sample A, a constant amount of 5 μg of total RNA was used to perform retrotranscription. RNA was quantified at 260 nm with Nanodrop (Thermo Scientific®). RNA quality was checked on Agilent 2100 Bioanalyzer (Agilent technologies®). Synthesis of radiolabelled cDNA, nylon array hybridization and washings were carried out as described previously [22]. Microarrays (from A to H) were exposed to a phospho-imager screen for eight days and scanned with a phospho-fluoroimager (Storm 860, Molecular Dynamics®). Validation of this transcriptomic protocol in *L. lactis* by qRT-PCR was previously provided [47]. Three series of eight microarrays from A to H were obtained from the three independent polysomal profile determinations. For each gene, spotted twice on the microarrays, the mean of the two intensities after background removal was considered. For each microarray (from A to H), a cutoff value was defined as the mean intensities of the “empty” spots plus one standard deviation as previously described [22]. 1619 genes presenting at least a mean intensity above the cutoff value for one of the eight microarrays were selected for further analysis.

For each microarray series, intra-series normalization to correct experimental variations after fraction collection was performed using a common reference. Each gene signal intensity of microarrays B to H was standardized by the mean intensity of reference microarray A (unfractionated RNA). We noted ${\left(I_{gene_{i,k}}^{Array_{j}}\right)}_{\mathrm{normArrayA}}$ the normalized intensity I of the gene *i* ∈ {1, …, 1619} that belonged to microarray *j* ∈ {*B*, *C*, *D*, *E*, *F*, *G*, *H*} by microarray A, for series replicates *k* ∈ {1, 2, 3}.

(2) $${\left(I_{gene_{i,k}}^{Array_{j}}\right)}_{\mathrm{normArrayA}}=\frac{I_{gene_{i,k}}^{Array_{j}}}{\sum_{i=1}^{i=1619}\left(I_{gene_{i,k}}^{Array_{A}}\right)}\times 1619$$

with

$$j\in \left\{B,C,D,E,F,G,H\right\},i\in \left\{1,\ldots ,1619\right\},k\in \left\{1,2,3\right\}$$

In order to take into account the variability in total RNA amounts between fractions B to H (from 29.7 μg ± 24.3 to 436.3 μg ± 170.3) of the same polysomal profile determination, and thus to work with RNA concentrations in the fraction instead of abundances, we corrected intensity values by total RNA quantity and named these intensities N (Equation 3).

(3) $$N_{gene_{i,k}}^{Array_{j}}={\left(I_{gene_{i,k}}^{Array_{j}}\right)}_{\mathrm{normArrayA}}\times \frac{totalRNA\;quantity_{k}^{Array_{j}}}{totalRNA\;quantity_{k}^{Array_{A}}}$$

For each microarray from B to H, an inter-series normalization step was introduced to adjust the signals of the three triplicates. For each microarray, an average intensity set was calculated from the three repetitions. The intensities of each repetition were plotted *versus* the average intensity set. From each plot, we estimated a linear regression coefficient *r* and an intercept coefficient *b* (Equation 4)

(4) $$N_{gene_{i,k}}^{Array_{j}}=r_{j,k}\times \left({\overset{Â¯}{N}}_{gene_{i}}^{Array_{j}}\right)+b_{j,k}+\xi_{k,i,j}$$

Where ${\overset{Â¯}{N}}_{gene_{i}}^{Array_{j}}$ denoted the mean of the three replications.

Those estimations were denoted respectively ${\hat{r}}_{j,k}$ and ${\hat{b}}_{j,k}\text{.}$

The intensities of each repetition were then centered with their own *b* coefficient and reduced by their own *r* coefficient. We noted the resulting normalized intensity as ${\left(N_{gene_{i,k}}^{Array_{j}}\right)}^{\mathrm{CR}}$ (Equation 5):

(5) $${\left(N_{gene_{i,k}}^{Array_{j}}\right)}^{\mathrm{CR}}=\frac{\left(N_{gene_{i,k}}^{Array_{j}}-{\hat{b}}_{j,k}\right)}{{\hat{r}}_{j,k}}$$

### Translatome variable calculations

For the 1619 genes with signal intensities above the cutoff values, two translatome variables were calculated, their ribosome occupancy and ribosome density.

For each gene, we calculated the proportions of mRNA molecules in each fraction from B to H:

(6) $$mRNA\;proportion_{gene_{i,k}}^{Array_{j}}=\frac{{\left(N_{gene_{i,k}}^{Array_{j}}\right)}^{\mathrm{CR}}}{\sum_{j=B}^{j=H}{\left(N_{gene_{i,k}}^{Array_{j}}\right)}^{\mathrm{CR}}}$$

For each gene, ribosome occupancy is the fraction of its mRNA population engaged in translation. It is calculated by summing the proportions of its mRNAs in fractions D to H (Equation 7). In fractions B and C, mRNA molecules were free or associated with an incomplete ribosome, so these mRNAs were not considered as engaged in translation.

(7) $$Ribosome\;occupancy_{gene_{i,k}}=\sum_{j=D}^{j=H}mRNA\;proportion_{gene_{i,k}}^{Array_{j}}$$

For each gene, the three ribosome occupancy values obtained for each series were averaged (Equation 8).

(8) $$\overset{Â¯}{Ribosome\;occupancy_{gene_{i}}}=\frac{1}{3}\sum_{k=1}^{k=3}Ribosome\;occupancy_{gene_{i,k}}$$

For each gene, the peak fraction corresponds to the highest mRNA proportion within fractions D to H containing mRNA engaged in translation. The peak fraction was determined by a bootstrap method on residuals. This procedure has already been used in transcriptome analysis and allowed increased robustness of the results [48]. This method does not require any assumptions on data distribution and corresponds to a resampling procedure with replacement. The residuals ε_*i,k*_ from each average value, from all seven fractions, were calculated (Equation 9):

(9) $$\varepsilon_{i,k}=mRNA\;proportion_{gene_{i,k}}-\overset{Â¯}{mRNA\;proportion_{gene_{i}}}$$

Then residuals were pooled together and reassigned back to these fractions at random to create a bootstrap data set. More precisely, for each gene *i* and for each value of *k*, a value of residual was sampled in the pool of residuals and was denoted by ${\tilde{\varepsilon }}_{i,k}$. This residual was added to the mean of the mRNA proportions of the gene *i* in order to create a bootstrap value of mRNA proportion (Equation 10).

(10) $$mRNA\;proportion{\;}_{gene_{i,kbootstrap}}=\overset{Â¯}{mRNA\;proportion_{gene_{i}}}+{\tilde{\varepsilon }}_{i,k}$$

Ten thousand bootstrap data sets were made. The peak fraction was determined in each bootstrap data set analogously to the initial data set. From the ten thousand bootstrap data sets, the relative frequency of the highest mRNA proportion was calculated with a confidence interval fixed at 95%. When the 95% bootstrap confidence interval was not confined to a single fraction, the definition of the peak fraction was widened from only one to two (or more) adjacent fractions and a search for the maximum was initiated again.

From a set of 1619 genes, a peak fraction was assigned to 1177 genes: all genes had a peak fraction contained in a single fraction. For each of the 1177 genes, we calculated the ribosome density that was the number of bound ribosomes in the peak fraction normalized with respect to the transcript length. The experimental length of all transcripts was not available in *L. lactis* and predictions were not considered to be confident [49]. Thus, we used the coding sequence length instead of transcript length in our calculation:

(11) $$Ribosome\;density_{gene_{i}}=\frac{ribosome\;number\;of\;the\;Peak\;Fraction_{gene_{i}}}{Coding\;Sequence\;length_{gene_{i}}}$$

Ribosome density and ribosome occupancy values are available in Additional file 4: Table S1.

### Enrichment analysis

In a given gene subset, statistical testing of the enrichment of genes having a characteristic of interest was performed. In a general way, if it is assumed that n_1_ genes were sampled without replacement in a total group of n_2_ genes, m of which have the characteristic of interest, the number N of genes having the characteristic of interest in the subgroup of n_1_ genes follows the hypergeometric distribution: N~H(m, (n_2_-m), n_1_). The *p*-value of the enrichment test of genes having the characteristic of interest in the n_1_ gene subgroup is defined as follows:

(12) $$p-value=P\left(N>N_{\mathrm{obs}}\right)$$

where N_obs_ is the observed value of N.

The *p*-value was calculated using R software.

### Covariance model

A linear analysis of covariance model was used to identify the major determinants of three variables of interest, namely ribosome occupancy, ribosome density and protein level. To do so, each model was established from various quantitative and qualitative parameters as described previously [3]. In a previous study [3], gene parameters such as chromosomal position, open reading frame length, CAI, gene functional category, protein hydrophobicity (Gravy score) and aromaticity have already been described, and mRNA and protein concentrations were provided. mRNA half-life measurements are from [22]. The upstream mRNA sequences (from −100 to +24 bp relative to the start codon) and downstream sequences (from −24 to +100 bp relative to the stop codon) were obtained from RSAtools and then processed with RNAfold software ( http://mobyle.pasteur.fr/cgi-bin/portal.py) specifying a temperature of 30°C. For each sequence, we used the free energy of the predicted minimum free energy structure (the most negative ΔG, ΔGup in the upstream sequence and ΔGdown in the downstream sequence) as a measure of secondary structure formation. Quantitative parameters were log-transformed to obtain a normal distribution except for those parameters which can take negative values (position, Gravy score and folding energy) and all were centered and reduced. This normalization was applied in order to adjust their level and allow comparison of model coefficients. For example, Equation 13 described the models established to explain the two translatome variables.

(13) $$\begin{array}{l} \ln\left(ribosome\;occupancy\left(i\right)\right)\;\text{or}\ln\left(ribosome\;density\left(i\right)\right)\\ \;=\alpha +{\beta_{Chrom.location}}_{\left(i\right)}\ln\left(Chrom.location_{\left(i\right)}\right)\\ \;+{\beta_{\left[mRNA\right]}}_{\left(i\right)}\ln\left({\left[mRNA\right]}_{\left(i\right)}\right)\\ \;+\beta_{mRN{A_{t1/2}}_{\left(i\right)}}\ln\left(mRN{A_{t1/2}}_{\left(i\right)}\right)\\ \;+{\beta_{CDS\;length}}_{\left(i\right)}\ln\left(CDS\;length_{\left(i\right)}\right)\\ \;+{\beta_{\mathrm{arom}}}_{\left(i\right)}\ln\left(arom_{\left(i\right)}\right)+{\beta_{\mathrm{hydro}}}_{\left(i\right)}\ln\left(hydro_{\left(i\right)}\right)\\ \;+{\beta_{\mathrm{CAI}}}_{\left(i\right)}\ln\left(CAI_{\left(i\right)}\right)+\beta_{\mathrm{\Delta G}}{{}_{\mathrm{up}}}_{\left(i\right)}\ln\left(\Delta {G_{\mathrm{up}}}_{\left(i\right)}\right)\\ \;+\beta_{\mathrm{\Delta G}}{{}_{\mathrm{down}}}_{\left(i\right)}\ln\left(\Delta {G_{\mathrm{down}}}_{\left(i\right)}\right)+\lambda_{cat_{\left(i\right)}}+\xi_{\left(i\right)} \end{array}$$

where *ribosome occupancy*_*(i)*_ and *ribosome density*_*(i)*_ are the measured levels of the *i*th value for the variable of interest, α is the intercept, *parameter j*_(*i*)_ the value of quantitative parameter j for the *i*th value, β_j(i)_ and λ_k(i)_ the coefficients associated to the *j*th quantitative parameter and the *k*th qualitative parameter of the *i*th value, respectively, and ζ_(i)_ the error term for the *i*th value. Parameter abbreviations used are: *Chrom.location* for chromosome location, *[mRNA]* for mRNA concentration, *mRNAt*_*1/2*_ for mRNA half-life, *arom* for aromaticity, *hydro* for hydrophobicity, *CAI* for codon adaptation index, *ΔG*_*up*_ for minimum free energy structure in the upstream sequence, *ΔG*_*down*_ for minimum free energy structure in the downstream sequence, *cat* for functional category. The model to explain the variable protein concentration was similar to that described above except that both ribosome density and ribosome occupancy were added as explanatory parameters. For each model, we obtained an estimate of the variable of interest. Least squares procedure was used to estimate coefficients of selected parameters and quality of modeling adjustment was obtained by calculation of the determination coefficient. The Akaike Information Criterion was used to select the best models without any *a priori* subjective parameter selection [3]. Adjusted r-squared values of our models were lower than 0.60 indicating that additional major explanatory parameters need still to be identified.

### Simple linear correlation

Simple correlations were estimated by calculating Pearson correlation coefficient and associated *p*-value using R free statistical software.

### Motif research

The presence of sequence motifs was explored using MEME suite software, section MEME ( http://meme.nbcr.net) and confirmed by RSAtools software ( http://rsat.ulb.ac.be/rsat/), section oligoanalysis with a default parameter selection for 5, 6 or 8 nucleotide lengths. The nucleotide sequences in the vicinity of the start codon (from −100 to +24 and −30 to +24 relatives to ATG) were also obtained from RSAtools (retrieve sequence section, default parameters).

## Competing interests

The authors declare that they have no competing interests.

## Authors' contributions

FP, PL, MC-B, LG: conception, data acquisition, analysis of the data, drafting of the manuscript; FP, HM, BL, LG, MC–B: statistical treatment of the data. All authors read and approved the final manuscript.

## Supplementary Material

Additional file 1 **Figure S3.** Ribosome density and ribosome occupancy values of *L. lactis* cells grown in exponential phase. Click here for file

Additional file 2 **Table S2.** Simple correlation analyses. Pearson correlation coefficients and the associated *p*-value were calculated. Click here for file

Additional file 3 **Figure S4.** Dot plot of ribosome occupancy *versus* ribosome density of genes involved in metabolic pathways discussed in the text. The dashed line indicates the ribosome density mean value of 1.31 ribosomes per 100 nucleotides calculated when considering the entire set of 1049 genes with a ribosome density value. The dotted line shows the ribosome occupancy mean value of 67% obtained for the1619 gene set with a ribosome occupancy value. Click here for file

Additional file 4 **Table S1.** Correlation between ribosome density and CDS length for the 814 genes used in the modeling approach. The upper part of the curve entitled ”high” corresponds to heavily loaded-ribosome genes (peak fraction in fraction H; 9.6-17.9 loaded ribosomes per transcript) while the lower part corresponds to genes loaded with only one ribosome (peak fraction in the monosome fraction D). Click here for file
