# Supplementary material for: Bacterial translational regulations: high diversity between all mRNAs and major role in gene expression
Source: BMC Genomics. 2012 Oct 4;13:528. doi: 10.1186/1471-2164-13-528 (PMC3543184; doi:10.1186/1471-2164-13-528)
Supplement: Additional file 2 — Table S2. Simple correlation analyses. Pearson correlation coefficients and the associated p-value were calculated. [file 1471-2164-13-528-S2.doc]

**Table S2 -Simple correlation analysis**. Pearson correlation coefficients and the associated *p*-value were calculated.

| Parameter | Ribosome occupancy | Ribosome density |
| --- | --- | --- |
| mRNA concentration | 0.16 (*p* = 3.11 x 10^-6^) | -0.14 (*p* = 7.77 x 10^-5^) |
| mRNA half-life | -0.44 (*p* = 5.75 x 10^-40^) | -0.27 (*p* = 2.61 x 10^-15^) |
| CDS length | 0.30 (*p* <2.0 x 10^-16^) | -0.14 (*p* = 3.94 x 10^-5^) |
| Aromaticity | / | 0.13 (*p* = 2.06 x 10^-4^) |
| CAI | 0.20 (*p* =1.74 x 10^-8^) | -0.16 (*p* = 3.79 x 10^-6^) |
| ΔGdown | / | 0.15 (*p* = 1.46 x 10^-5^) |
| ΔGup | / | 0.15 (*p* = 1.14 x 10^-5^) |
