# Supplementary figures and images for: Bacterial translational regulations: high diversity between all mRNAs and major role in gene expression
Source: BMC Genomics. 2012 Oct 4;13:528. doi: 10.1186/1471-2164-13-528 (PMC3543184; doi:10.1186/1471-2164-13-528)

## Slide 1
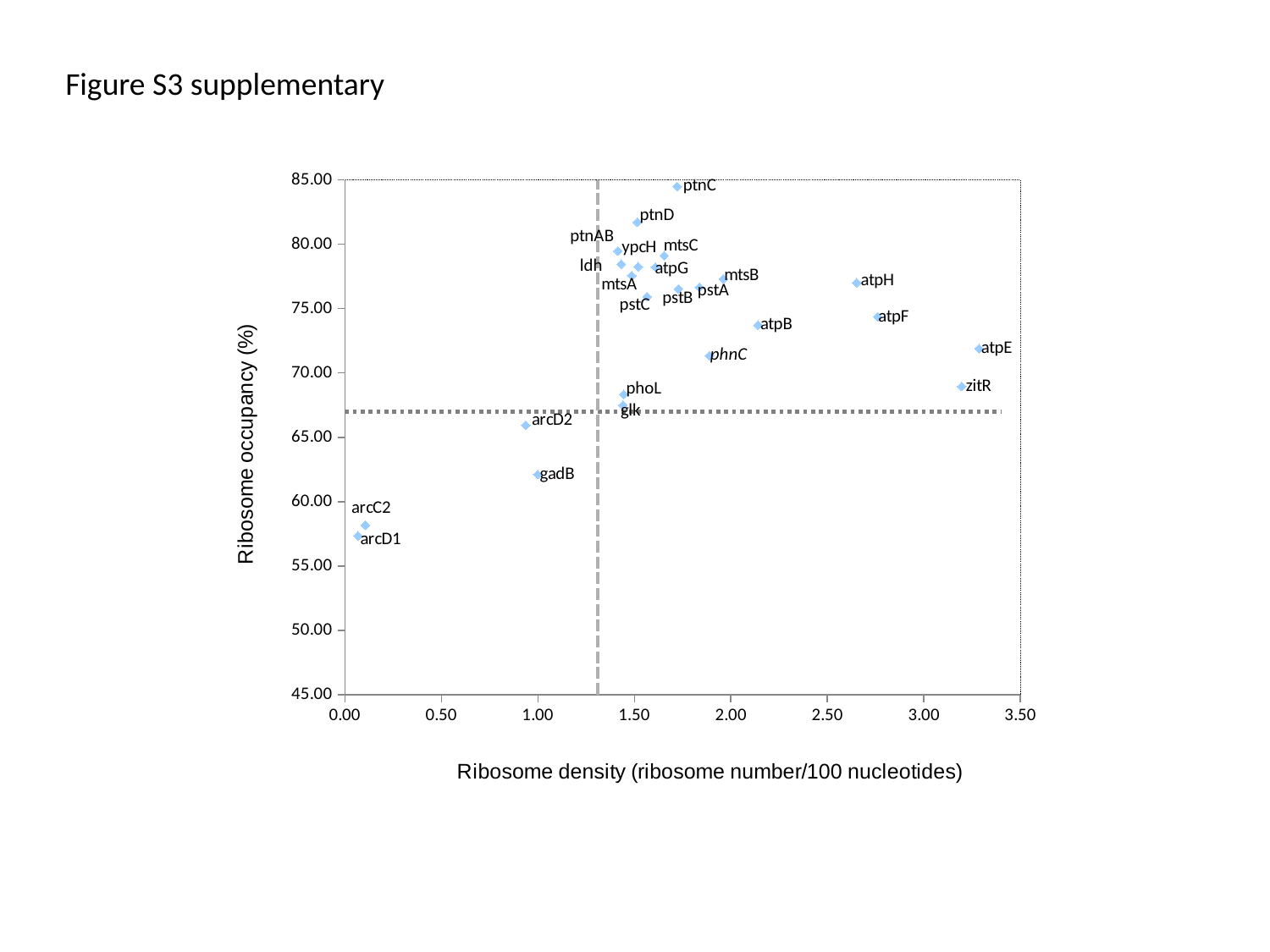

Figure S3 supplementary
### Chart
| Category | | | |
|---|---|---|---|

Supplement: Additional file 3 — Figure S4. Dot plot of ribosome occupancy versus ribosome density of genes involved in metabolic pathways discussed in the text. The dashed line indicates the ribosome density mean value of 1.31 ribosomes per 100 nucleotides calculated when considering the entire set of 1049 genes with a ribosome density value. The dotted line shows the ribosome occupancy mean value of 67% obtained for the1619 gene set with a ribosome occupancy value. [file 1471-2164-13-528-S3.pptx]

## Slide 1
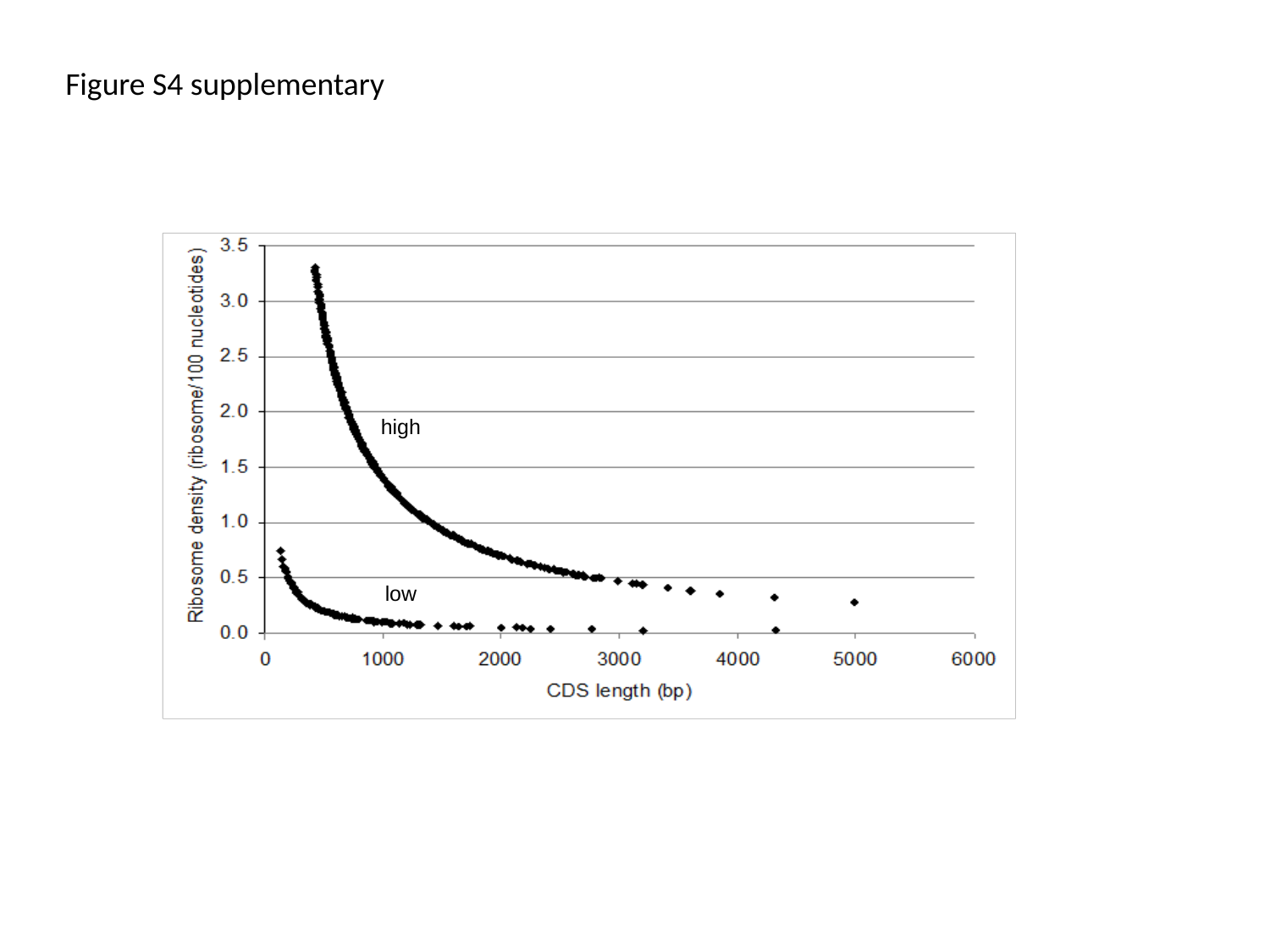

Figure S4 supplementary
high
low

Supplement: Additional file 4 — Table S1. Correlation between ribosome density and CDS length for the 814 genes used in the modeling approach. The upper part of the curve entitled ”high” corresponds to heavily loaded-ribosome genes (peak fraction in fraction H; 9.6-17.9 loaded ribosomes per transcript) while the lower part corresponds to genes loaded with only one ribosome (peak fraction in the monosome fraction D). [file 1471-2164-13-528-S4.pptx]
